# The use of mechanical circulatory support in elective high-risk percutaneous coronary interventions: a literature-based review

**DOI:** 10.1093/ehjopen/oeae007

**Published:** 2024-02-09

**Authors:** Alexander Geppert, Kambis Mashayekhi, Kurt Huber

**Affiliations:** 3rd Department of Medicine, Cardiology and Intensive Care Medicine, Clinic Ottakring, Montleartstrasse 37, A-1160 Vienna, Austria; Division of Cardiology and Angiology II, University Heart Center Freiburg—Bad Krozingen, Südring 15, D-79189 Bad Krozingen, Faculty of Medicine of the University, Freiburg, Germany; Clinic of Internal Medicine and Cardiology, Heart Center Lahr, Hohbergweg 2, D-77933 Lahr/Schwarzwald, Germany; 3rd Department of Medicine, Cardiology and Intensive Care Medicine, Clinic Ottakring, Montleartstrasse 37, A-1160 Vienna, Austria; Medical Faculty, Sigmund Freud University, Freudplatz 1+3, A-1020 Vienna, Austria

**Keywords:** Mechanical circulatory support, High-risk percutaneous coronary intervention, IABP, Impella, V-A ECMO

## Abstract

Contemporary medical practices allow complete percutaneous coronary intervention (PCI) in a considerable number of patients who previously would have been considered too ‘high-risk’ for such procedures. The use of mechanical circulatory support (MCS) devices during these high-risk PCIs (HR-PCIs) is thought to reduce the potential risk for major adverse events during and after revascularization. The intra-aortic balloon pump (IABP), veno-arterial extracorporeal membrane oxygenation (V-A ECMO), and the Impella are the most common MCS devices in use. This review aims to summarize the clinical evidence for each of these devices and the potential mechanisms for the improvement in patient outcomes in HR-PCI. The IABP use has rapidly declined in recent years due to no evidence of benefit in HR-PCI and cardiogenic shock. The V-A ECMO results in low rates of major adverse cardiac and cerebrovascular events (MACCEs) but higher rates of acute kidney injury and increased need for transfusions. In initial studies, Impella resulted in a reduced need for repeat interventions and reduced rates of hypotension, but no benefit in mortality. However, MACCE rates with Impella have gradually declined over the last 10 years, reflecting increased operator experience and technical improvements. Thus, a large, randomized trial is needed to assess the efficacy of Impella in HR-PCI with contemporary standards of care. There is currently no individual parameter that can identify patients who would benefit from MCS use in elective HR-PCI. To address this gap, we propose an algorithm that combines anatomical complexity, comorbidities, and clinical presentation to accurately identify candidates for MCS-assisted HR-PCI.

## Introduction

In stable patients with multivessel disease, left main disease or complex anatomy, and reduced left ventricular ejection fraction (LVEF), coronary artery bypass grafting (CABG) provides better outcomes than medical therapy or percutaneous coronary intervention (PCI).^1,2^ Particularly, patients with an intermediate-to-high SYNTAX score seem to benefit from surgical revascularization.^3^ In addition to PCI decisions guided by SYNTAX score, there are several other classes of patients deemed inoperable or of unacceptable high-surgical risk that are potential candidates for PCI. This includes patients with poor targets for bypass grafts, patients after a CABG procedure with an open left internal mammary artery, and patients with a porcelain aorta, frailty, or severe pulmonary or cerebrovascular disease. As outlined in the recently presented OPTIMUM study,^4^ these patients not only present with severe clinical risk factors but also a high procedural risk with 45% having a SYNTAX score >33, 80% with heavy calcification, and 56% with a chronic total occlusion. If treated by PCI, these patients usually need complex, long-lasting interventions with special procedural considerations. Consequently, they are at high risk of major adverse cardiac and cerebrovascular events (MACCEs) during or after PCI, classifying procedures such as high-risk percutaneous coronary interventions (HR-PCIs). At present, there is no unifying definition of HR-PCI, although there is a consensus that clinical-, patient-, and lesion-specific factors must be considered.^5,6^ To facilitate treatment and improve outcomes in these challenging cases, several new or refined interventional techniques and percutaneous mechanical circulatory support (MCS) devices have been developed over the years in an attempt to reduce and eliminate PCI complications. In this review, we will give a technical overview of the three most popular MCS devices: the intra-aortic balloon pump (IABP), veno-arterial extracorporeal membrane oxygenation (V-A ECMO), and Impella. Additionally, we outline potential mechanisms for MCS-related improvement in patient outcomes after HR-PCI and discuss the available evidence for each of the three devices. Finally, we will suggest a new algorithm for identifying patients who may benefit from MCS use in HR-PCI. Notably, patients with acute heart failure or cardiogenic shock (CS) constitute a separate group of patients with different needs and therefore will not be discussed here.

### Technical overview of mechanical circulatory support devices

**Figure 1:**
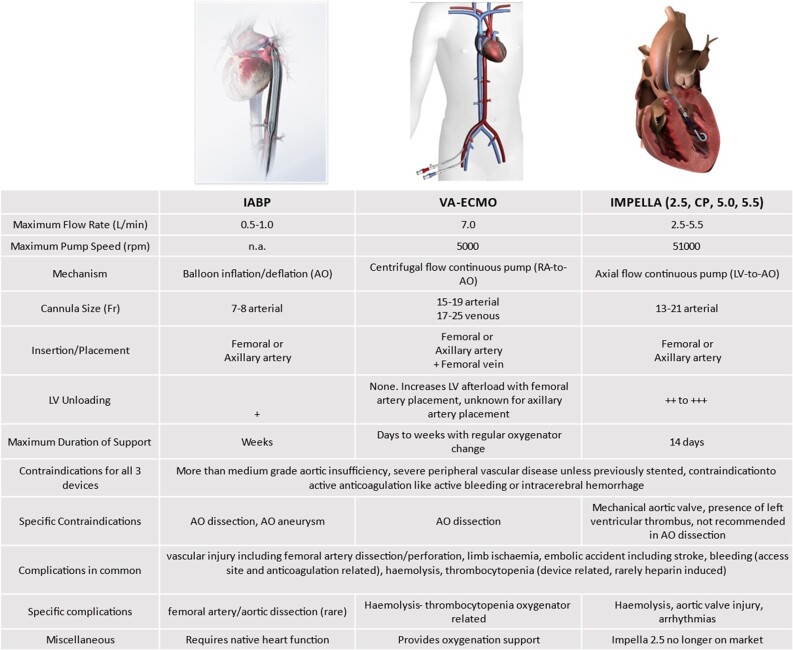
Mode of action of different mechanical circulatory support with a maximum duration of support, explicit contraindications, and possible complications. It should be noted that the use of heparin-coated systems allows veno-arterial extracorporeal membrane oxygenation systems to be operated for several hours without systemic anticoagulation. AO, aorta; IABP, intra-aortic balloon pump; LV, left ventricle; RA, right atrium; V-A ECMO, veno-arterial extracorporeal membrane oxygenation. Pictures of IABP and ECMO were provided by Getinge Deutschland GmbH, Rastatt, Germany and the picture from Impella was provided by Abiomed Europe GmbH, Aachen, Germany.

The most common MCS systems used in HR-PCI are IABP (multiple companies), ECMO (multiple companies), and Impella (Abiomed).^7^ Detailed characteristics have been summarized previously.^6^ For this review, we will briefly describe each of the three devices and summarize the implantation site, its effect on cardiac output (CO), left ventricular (LV) unloading, and relevant contraindications (*Figure 1*).

#### The intra-aortic balloon pump

First used in 1967, the IABP utilizes pulse-synchronized inflation and deflation of a balloon in the descending aorta. The diastolic augmentation associated with balloon inflation during diastole increases diastolic pressure and, consequently, mean arterial pressure. Deflation of the balloon during systole causes a decrease in LV afterload, resulting in LV unloading and a modest increase in CO (0.5 L/min). Taken together, this leads to augmented coronary and systemic perfusion^8^ and improved oxygen consumption of the heart.^9^ For decades, the IABP was the most widely implanted MCS device in HR-PCI due to its ease of implantation and simplicity of use. Possible complications and contraindications are summarized in *Figure 1*.

#### Extracorporeal membrane oxygenation

Extracorporeal membrane oxygenation is a device that actively drains blood from the venous system, oxygenates it, removes carbon dioxide, and returns blood to the circulatory system. ECMO can be implemented in a veno-venous (V-V) or veno-arterial (V-A) configuration, returning blood to the venous or arterial circulations, respectively. Veno-venous extracorporeal membrane oxygenation is used for pulmonary failure and V-A ECMO for circulatory failure or HR-PCI. The device consists of a centrifugal pump, an oxygenator, and two cannulas in two veins (V-V ECMO) or a vein and an artery (V-A ECMO). For HR-PCI settings, the V-A ECMO is usually set up in a peripheral configuration (i.e. cannulation of the femoral vein and the common femoral artery).

Extracorporeal membrane oxygenation can provide continuous centrifugal flow up to 7 L/min and is largely dependent on venous inflow,^10^ but flow rates >5 L/min are seldom used due to the occurrence of haemolysis at high flow rates. The major advantages of V-A ECMO include the relative ease of implantation in emergencies without the explicit need for fluoroscopic guidance, the possibility of extended support, oxygenation, and the capability to provide complete extracorporeal life support. However, it has several disadvantages. If V-A ECMO is set up in the peripheral configuration, this causes an increase in afterload for the LV. This increase in afterload occurs independently of right ventricular (RV) function. As such, lung congestion/pulmonary oedema can occur especially when the RV grants an adequate output. Additionally, the large extracorporeal circuit introduces the potential for coagulation and inflammation, despite biocompatible coatings. Finally, the large cannulas increase the risk of bleeding complications and limb ischaemia; therefore, an antegrade limb perfusion cannula on the side of the arterial cannula is recommended. Possible complications and contraindications for V-A ECMO are provided in *Figure 1*.

#### The Impella device

The Impella device was released in the USA in 2008 and in Europe in 2012. It is a microaxial flow pump that is inserted through the femoral or subclavian/axillary artery. Femoral implantation can be accomplished with percutaneous implantation, whereas subclavian/axillary placement and placement of the larger pumps via the femoral usually require surgical implantation. The Impella device is advanced across the aortic valve into the LV with fluoroscopic guidance. Once in place, the pump actively transfers blood directly from the LV to the ascending aorta, increasing CO, aortic, and coronary perfusion pressures. It is particularly beneficial when PCI causes a decrease in CO and blood pressure. In addition, the Impella decreases left ventricular end-diastolic pressure (LVEDP), thereby relieving LV systolic and diastolic wall stress and improving microvascular perfusion.

Initial implementations of the Impella pump offered a maximum of 2.5 or 5 L/min LV output. In 2016, the Impella CP was first introduced, which offers a maximum of 4.3 L/min LV output and can be implanted percutaneously via a 14 Fr sheath. Implantation of Impella 2.5 required insertion of a 12 Fr sheath and could be performed percutaneously through the femoral artery. The stiffer, Impella 5.0, necessitated a 21 Fr sheath and could only be implanted surgically. Moreover, the Impella CP provides comparable support to both ECMO and the Impella 5.0 if these devices are run at moderate flow rates. As such, Impella devices received initial approval for extended use up to 14 days in 2019, and CE marking in 2018 for use up to 4–6 days for HR-PCI. Collectively, the major advantages of the Impella devices are greater haemodynamic support compared with the IABP, and active unloading of the LV compared with both IABP and V-A ECMO. Major disadvantages of Impella include the lack of active oxygenation and the need for adequate RV output to provide adequate LV filling. In fact, the latter is a major determinant for the generation of efficient CO by the Impella device. Consequently, this is a major limitation of Impella making it less efficient in prolonged cardiac arrest situations, including arrhythmic storms. Possible complications and contraindications for the use of Impella are summarized in *Figure 1*.

Increased MCS device use and a greater understanding of the limitations of ECMO and Impella have led to the simultaneous implementation of both techniques, termed ECPELLA. ECPELLA is implemented to improve oxygenation and unloading of the LV in patients with more severe heart conditions, thus overcoming the limitations of each device and providing improved patient outcomes.

### Impact of mechanical circulatory support device use on complete revascularization

In lengthy and complicated HR-PCI procedures, notably in patients with LVEF <35%, MCS devices are implemented to reduce immediate peri-procedural complications such as acute left heart failure with severe haemodynamic compromise leading to CS with potential electrical instability and respiratory failure. The use of MCS devices in HR-PCI may, however, also reduce mid- and long-term MACCE. Potential mechanisms for improvement in patient outcomes are discussed below, although none have been examined in randomized clinical studies.

#### Reduction of mid- and long-term major adverse cardiac and cerebrovascular event by complete anatomical and/or functional revascularization

Mechanical circulatory support devices may improve patient outcomes by promoting complete revascularization of all angiographically significant lesions.^11,12^ The RESTORE EF study,^13^ a multicentre, non-randomized trial of 406 patients in the USA treated with Impella during elective HR-PCI, reported increased LVEF in patients with complete revascularization after 90 days, with the greatest improvement in patients with a residual SYNTAX score of 0 or LVEF <20% at baseline. Moreover, recent studies, including a meta-analysis of over 17 000 patients, have demonstrated that PCI guided by intravascular imaging (via ultrasound or optical coherence tomography) is associated with improved patient outcomes.^14–16^ A recently published randomized trial demonstrated that image-guided PCI in complex coronary procedures is even superior in terms of hard clinical endpoints.^17^ The additional use of MCS devices during HR-PCI might provide the necessary support to prevent peri-procedural events during protracted interventions. This may especially be the case with complex procedures like intravascular lithotripsy or rotablation/rotational atherectomy, as well as during post-stent optimization triggered by meticulous lesion assessment using intravascular imaging. Thus, by promoting optimal revascularization, MCS might reduce the risks of restenosis, stent thrombosis, and subsequent revascularization. Notably, complete revascularization has been shown to occur more often with Impella than with IABP.^18^ In the PROTECT II study,^18^ the use of rotational atherectomy was 9% in the IABP group and 14.2% in the Impella group, which was further increased to 52% in the RESTORE EF population.^13^ However, the use of MCS devices has a higher rate of vascular bleeding and is known to be a predictor for in-hospital MACCE.^19^

The use of MCS devices may also facilitate PCI of all functionally relevant stenoses in one procedure. Indeed, intermediate, non-critical lesions are often difficult to assess by angiography or intravascular imaging alone. The haemodynamic stability during HR-PCI with MCS devices might also allow repeated physiological measurements with hyperaemic agents to identify all relevant lesions even during extended procedures in patients with severely reduced LVEF (<35%) and therefore could reduce the rate of repeat interventions. Accordingly, the FAME-2 trial demonstrated leaving functionally relevant lesions untreated, resulting in a higher rate of repeat interventions.^20^

### Clinical evidence of different mechanical circulatory support devices during high-risk percutaneous coronary intervention

#### Intra-aortic balloon pump in high-risk percutaneous coronary intervention

The BCIS-1 prospective, open, multicentre, randomized controlled trial published in 2010 evaluated the efficacy of elective IABP therapy for reducing MACCE during HR-PCI.^21^ The control group consisted of patients without planned IABP use during HR-PCI.^21^ High-risk was defined as the presence of two factors: LVEF <30% and either a BCIS-1 Jeopardy score of ≥8, a left main coronary artery stenosis, or a target vessel that supplied ≥40% of the myocardium. While the MACCE rate at hospital discharge and over 6 months were similar between patients in the IABP and control groups,^21^ long-term mortality was reduced by 34% in the IABP group.^22^ Prolonged hypotension occurred more often in the patients without IABP use (10.7 vs. 1.3%, *P* < 0.001), with rescue IABP insertion required in 18 (12%) of these patients. This, however, did not translate to higher rates of myocardial infarction or increased 28-day mortality in this treatment arm.^21^ A major limitation of this study is that, unlike the SYNTAX score, the BCIS-Jeopardy score utilized does not account for the anatomical complexity of stenotic lesions. Therefore, the anatomical complexity of the lesions treated in the BCIS-1 study is unclear, prohibiting direct comparisons to newer studies that use the SYNTAX score. A meta-analysis of 11 studies published in 2013 that exclude rescue IABP patients failed to demonstrate a beneficial effect of planned IABP use on mortality or MACCE in HR-PCI.^23^ Accordingly, the recent European Association of Percutaneous Coronary Interventions (EAPCI)/Association for Acute Cardiovascular Care (ACVC) expert consensus document on percutaneous ventricular assist devices does not recommend IABP use in HR-PCI.^24^

#### Extracorporeal membrane oxygenation in high-risk percutaneous coronary intervention

Data regarding the elective use of V-A ECMO in HR-PCI are scarce. In total, there are only a few small single- or dual-centre retrospective studies, each enrolling no more than 12–31 patients.^25–28^ Together these data show that in HR-PCI, V-A ECMO is associated with low MACCE (14–17%) and overall low mortality rates (0–7%).^25,26,28^ Despite these data, the recent EAPCI/ACVC expert consensus document does not recommend the use of V-A ECMO in HR-PCI, which is likely a result of data scarcity.^24^

#### Impella in high-risk percutaneous coronary intervention: the PROTECT II study

The efficacy of the Impella device in HR-PCI has been tested in a large randomized study (PROTECT II trial),^18^ several large registries,^29,30^ and small non-randomized studies.^11,13,31^ In addition, there have been several *post hoc* studies on the PROTECT II data set that offer valuable insights into Impella use.^12,32,33^

The PROTECT II study^18^ is the only published large randomized study comparing outcomes following the use of two MCS devices (Impella 2.5 vs. IABP) in HR-PCI. The study planned to enrol 327 patients per treatment arm but was discontinued prematurely at the suggestion of the Data and Safety Monitoring Board. At its completion, the PROTECT II study included a total of 452 patients in the intention-to-treat analysis and 427 patients in the per-protocol population, with the major adverse event (MAE) rate at 30 days post-intervention as the primary endpoint. The MAE rates in the per-protocol population were similar between the two groups (34 vs. 42%, *P* = 0.092) at 30 days but significantly lower at 90 days in the Impella 2.5 group than that in the IABP group (40 vs. 51%, *P* = 0.023).^18^ Importantly, most MAEs occurred after discharge, and the difference was largely driven by reduced rates of repeat revascularization (8 vs. 4%) and myocardial infarction (14 vs. 12%) in the Impella arm.

However, it should be noted that no significant differences in MAE at 90 days were found in the intention-to-treat population. The major reasons for exclusion were protocol deviations regarding inclusion criteria (e.g. LVEF >35%, no three-vessel disease, or unprotected left main disease). Moreover, the PROTECT II study compared support with Impella 2.5 to support with IABP. Therefore, it is unclear whether the results including those of the sub-studies would apply to patients treated with the Impella CP, given that Impella 2.5 is no longer on the market. As such, Impella CP is currently the predominantly implanted Impella device during HR-PCI. Finally, the primary endpoint of the PROTECT II study included traditional MACCE and follow-up cardiac or vascular operations, acute kidney injury (AKI), severe intra-procedural hypotension, cardiopulmonary resuscitation (CPR), ventricular tachycardia, aortic insufficiency, and angiographic failure of PCI. This may partially explain why MAE rates were higher in the PROTECT II trial than in similar clinical studies.

However, even traditional 30-day MACCE rates were higher in the PROTECT II than in the BCIS-1 study, likely driven by a higher mortality rate at 30 days in the PROTECT II study. It is unclear why overall mortality rates were higher in PROTECT II, but it is worth noting that two-thirds of PROTECT II study patients were deemed inoperable by surgical consultants, while no such data are available for the BCIS-1 study.^18^ Later, in a sub-study of 325 PROTECT II patients with 3-vessel disease and LVEF <30%, MAE rates at 90 days were significantly lower in the Impella arm compared with the IABP arm (40 vs. 51%, *P* = 0.039).^32^ In this sub-study, IABP-treated patients also had a higher degree of hypotension than Impella-treated patients, with the largest difference in mean arterial blood pressure drop between the two treatment arms observed in patients with all three vessels treated.

#### Importance of extensive revascularization for outcome in Impella-supported high-risk percutaneous coronary intervention

In another PROTECT II sub-study,^12^ MAE, mortality, and repeat revascularization rates were significantly reduced in patients with more extensive revascularization, irrespective of treatment with Impella or IABP. Nevertheless, extensively revascularized patients treated by Impella had even lower MAE rates than patients treated with IABP (32 vs. 51%, *P* = 0.008), although SYNTAX score reductions were comparable. Similarly, more complete revascularization was associated with better outcomes in the Impella Italian (IMP-IT) registry.^11^ In 145 patients who underwent HR-PCI, those with lower residual Jeopardy scores after PCI had a lower incidence of myocardial infarction (*P* = 0.036) compared with patients with less extensive revascularization, emphasizing that the extent of revascularization is an important determinant for outcome improvement in HR-PCI when using MCS devices.

#### Real-life registries of Impella in high-risk percutaneous coronary intervention: the US-PELLA registry and PROTECT III study

Data from the US-PELLA registry report on patient outcomes in real-world clinical settings, which include several hundred patients who underwent elective HR-PCI with Impella support from 2007 to 2013 to corroborate the results of the PROTECT II randomized study.^30^ A comparison of US-PELLA registry patients that would have met enrolment criteria for the PROTECT II trial (*n* = 339) to matched patients of the Impella arm of PROTECT II (*n* = 216)^30^ revealed a comparable reduction in mortality rate (2.8 vs. 4.2%, *P* < 0.1) in US-PELLA PROTECT II-like patients despite the older age and larger relevant comorbidity burden of patients from the US-PELLA registry. Collectively, this analysis suggests that the patient outcomes observed in the PROTECT II trial likely apply to real-world clinical scenarios.

The most recent PROTECT III study included 1134 patients with severely depressed LVEF who underwent elective, non-emergent PCI with Impella at 45 sites between March 2017 and March 2020.^29^ This study confirmed US-PELLA registry findings, demonstrating more complete revascularization, decreased MACCE rates, fewer bleeding complications, and less rates of hypotension (*P* < 0.01 for all) in PROTECT III patients compared with matched PROTECT II patients receiving Impella support.^29^ These improved outcomes may be explained by several factors. Firstly, there was a major development in the device itself between PROTECT II and III. Higher flow rates became possible with the Impella CP, causing less haemodynamic compromise and could explain the reduced rates of hypotension and need for CPR during PCI in PROTECT III patients compared with matched PROTECT II patients (0.2 vs. 6.6%, *P* = 0.003). Secondly, operator experience with the Impella device has increased over time, allowing more appropriate handling of the device, and better management of complications. In addition, the introduction of new vessel closure devices and the standardization of implantation procedures likely result in a reduction in bleeding and vascular complications.^29^ Experience with the Impella device is further emphasized in a PROTECT II sub-study that reported a lower MAE rate in Impella patients after excluding outcomes from the first patient in each treatment arm.^33^ Thirdly, more complete revascularization with Impella support may improve LVEF, as evidenced in the PROTECT II study,^18^ the Roma-Verona study,^31^ and the RESTORE EF study.^13^ Finally, Impella may reduce AKI in patients with reduced LVEF and long procedure times compared with those not treated with an MCS device (5.2 vs. 27.8%, *P* < 0.001).^34,35^  *Table 1* summarizes the currently available evidence on Impella-assisted HR-PCI.

**Table 1:** Summary of available evidence on Impella-assisted high-risk percutaneous coronary intervention

	Type of study	Impella effect
O’Neill *et al*.^18^	Single randomized study (*n* = 452)	Significant reduction in 90-day MAE when compared with IABP
Kovacic *et al*.^32^	*Post hoc* analysis of PROTECT II (*n* = 325)	Less degree of hypotension in patients with 3VD when compared with IABP
O’Neill *et al*.^29^ Cohen *et al*.^30^	Registry data (PROTECT III) (*n* = 504 PROTECT II-like patients) and US-PELLA registry (*n* = 339 PROTECT II-like patients)	Less severe hypotension in Impella-treated patients nowadays than in PROTECT II
O’Neill *et al*.^18^	Single randomized study (*n* = 452)	Reduction in 90-day revascularization rate when compared with IABP
Burke *et al*.^12^	*Post hoc* analysis of PROTECT II (*n* = 389)	Lower MAE in extensive Revascularization when compared with IABP
Schweitzer *et al*.^36^	Registry single centre (*n* = 28)	Reduced rate of AKI in Impella-assisted HR-PCI when compared with ECMO-assisted HR-PCI
Flaherty *et al*.^35^ Flaherty *et al*.^34^	Registry single centre matched control (*n* = 125) + multicentre (*n* = 223)	Reduced rate of AKI in HR-PCI when compared with no support
Aurigemma *et al*.^11^	Registry (*n* = 297)	Improved outcome in Impella patients with more extensive revascularization
Burzotta *et al*.^31^ Wollmuth *et al*.^13^	Registries (*n* = 86 + 150)	Greater improvement of LVEF in Impella patients with more extensive revascularization

AKI, acute kidney injury; ECMO, extracorporeal membrane oxygenation; HR-PCI, high-risk percutaneous coronary intervention; IABP, intra-aortic balloon pump; LVEF, left ventricular ejection fraction; MAE, major adverse event; PCI, percutaneous coronary intervention; VD, vessel disease.

The PROTECT IV study (NCT04763200) is currently underway to expand our knowledge of how Impella affects outcomes in HR-PCI. For this randomized clinical trial, the target enrolment is 1252 patients with reduced LVEF, receiving Impella support or standard-of-care PCI with or without IABP. The primary endpoint for analysis is the composite rate of all-cause death, stroke, necessitation of durable LVAD implant or heart transplant, myocardial infarction, and cardiovascular hospitalization at 3 years after the intervention.

#### Veno-arterial extracorporeal membrane oxygenation vs. Impella in high-risk percutaneous coronary intervention

To our knowledge, there are only two studies that compare the use of V-A ECMO to the use of Impella in patients with HR-PCI. One small non-randomized study^36^ found that despite having similar Mehran risk scores (*P* = 0.55), patients supported by Impella (*n* = 17) had a significantly reduced incidence of AKI compared with V-A ECMO-supported patients (*n* = 11) (12 vs. 55%; *P* = 0.03). One possible explanation is that Impella maintains a greater amount of pulsatile flow than V-A ECMO, which is critical to maintaining renal cortical blood flow and adequate kidney function.^37^ Additionally, procedure times were shorter in the Impella group (*P* = 0.01) despite the comparable complexity of coronary anatomy in the two groups and lower transfusion needs of the Impella group (45 vs. 6%, *P* = 0.02). The latter was most likely due to a diluting effect of the priming solution for the ECMO circuit. Furthermore, increased vasopressor needs were observed in the V-A ECMO group when compared with the Impella group (36 vs. 6%, *P* = 0.062). Irrespective of these differences, other clinical outcomes and MACCE rates were similar between the two treatment arms.^36^ The second study is a small multicentre observational study comparing Impella CP (*n* = 27)-supported HR-PCI patients to V-A ECMO (*n* = 14)-supported HR-PCI patients. As such, no statistically significant differences in mortality, MACCE, or bleeding rates were reported.^38^ Taken together, HR-PCI patients supported by V-A ECMO require longer procedure times and might be associated with a higher incidence of AKI, higher vasopressor use, and higher RBC transfusion need in post-procedural care than those supported by the Impella system. Of note, the recent recommendation by the European Association of Percutaneous Cardiovascular Interventions and Association for Acute Cardiovascular Care to not use V-A ECMO during HR-PCI^24^ was not related to the above results, as the recommendation was not available at the time of publication of the expert consensus document.

##### Timing of mechanical circulatory support implantation in elective high-risk percutaneous coronary intervention

As discussed, various risk constellations help identify patients who could benefit from MCS. However, the choice of MCS device and/or implementation approach still relies on operator experience. Furthermore, whether the timing of Impella implantation is critical to desired outcomes remains unclear. Recent data suggest that the elective use of MCS devices is more useful than rescue implantation.^39^ Here, 971 patients from the catheter-based ventricular assist device (cVAD) registry who were undergoing HR-PCI with pre-planned Impella were compared with 57 patients from the cVAD and US-PELLA databases receiving rescue Impella support during HR-PCI. In-hospital mortality was significantly higher in patients receiving rescue Impella vs. pre-planned Impella (49 vs. 4%, *P* < 0.001 in all patients; 57.8 vs. 4.4%, *P* < 0.001 in the propensity score-matched patients). Additionally, rescue patients necessitated CPR (22.8 vs. 1.5%, *P* < 0.001) and vasopressors/inotropes administration (77.2 vs. 11.4%, *P* < 0.001) more often than pre-planned Impella patients. Moreover, 41.7% of rescue patients expired while on support or following the withdrawal of care compared with only 0.7% in the pre-planned elective group (*P* < 0.001). Similar results were reported in the IMP-IT registry for preprocedural insertion of Impella in HR-PCI^40^ regarding 1-year mortality. This was also true of a composite of mortality, re-hospitalization for heart failure and need for left ventricular assist device/heart transplantation in patients receiving planned vs. rescue IABP in HR-PCI,^41^ and those receiving treatment for chronic total occlusion of a major coronary artery.^42^

##### Movement towards identifying patients that would benefit from mechanical circulatory support in high-risk percutaneous coronary intervention

**Figure 2:**
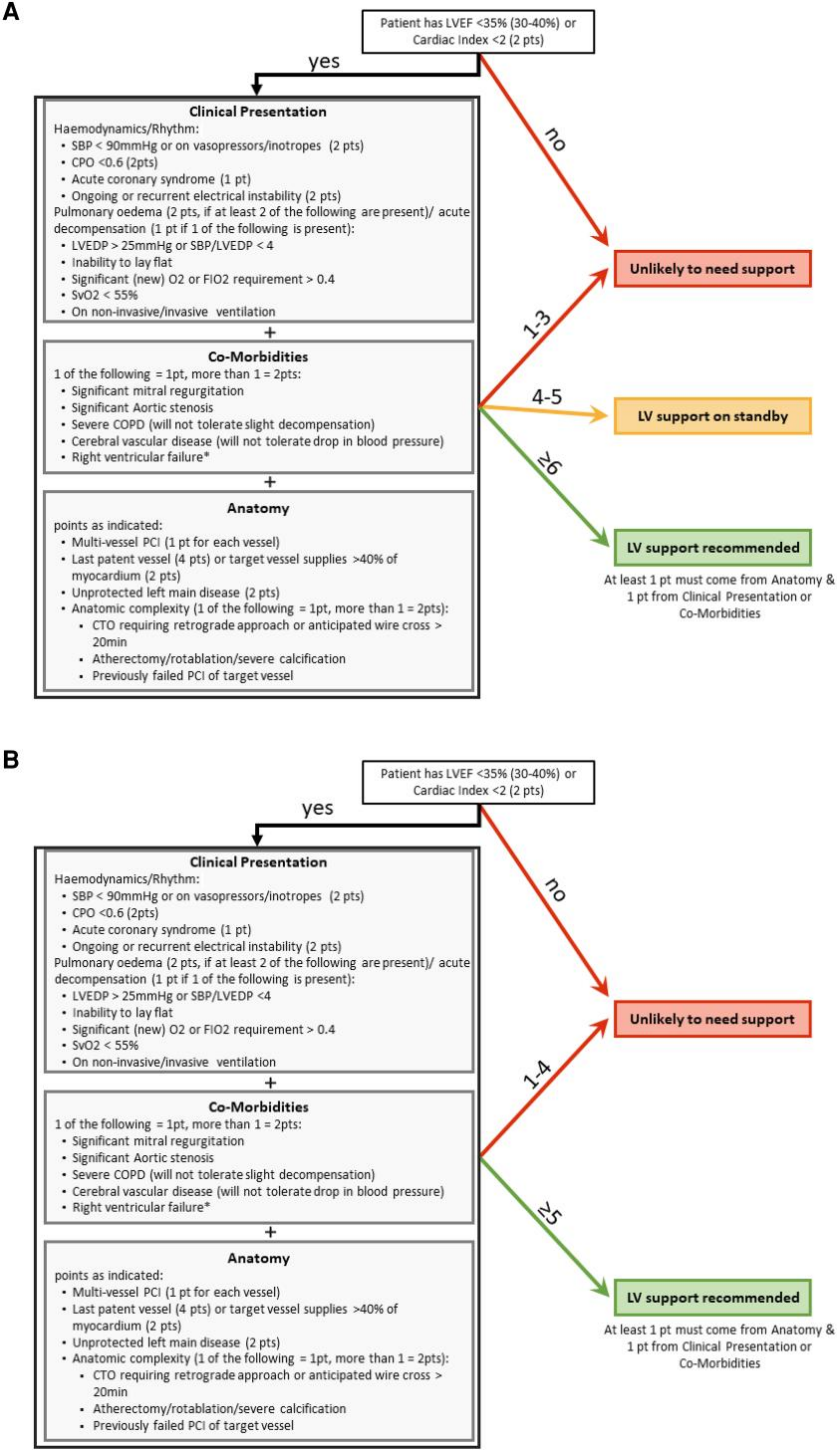
Proposed protected percutaneous coronary intervention algorithm. (*A*) Proposed protected percutaneous coronary intervention algorithm including the possibility for standby left ventricular support. (*B*) Proposed protected percutaneous coronary intervention algorithm not allowing the possibility of standby left ventricular support. Regardless, at least 1 point should come from ‘Anatomy’ because in our experience, non-complex stenoses can often be treated safely even in patients with severely depressed left ventricular ejection fraction and comorbidities without left ventricular support. Special consideration should be paid to the last patent vessel percutaneous coronary intervention (4 points assigned). Even with a normal left ventricular ejection fraction percutaneous coronary intervention, this situation might better be performed under the support of mechanical circulatory support. It should be noted that right ventricular failure is not to be treated with left-sided Impella support. The term ‘right ventricular failure’ here solely indicates that the concomitant presence of right ventricular failure increases the risk for high-risk percutaneous coronary intervention. COPD, chronic obstructive pulmonary disease; CTO, chronic total occlusion; FIO_2_, fraction of inspired oxygen; LVEDP, left ventricular end-diastolic pressure; LVEF, left ventricular ejection fraction; O_2_, oxygen; SBP, systolic blood pressure; SvO_2_, venous oxygen saturation; CPO, cardiac power output.

Unfortunately, unlike for CS patients, there is currently no individual parameter(s) that identifies patients at risk for haemodynamic compromise during elective HR-PCI.^43^ To better identify patients who would benefit from MCS use during elective HR-PCI, we suggest that a set of multiple variables generating an overall risk score should be used rather than individual parameters like LVEF and SYNTAX score. For instance, parameters relating to comorbidities, such as chronic obstructive pulmonary disease, cerebrovascular disease, and valvular heart disease have been infrequently reported in available studies but may also help identify patients in need of MCS. Of course, any slight left-sided heart failure during HR-PCI in a patient with chronic obstructive pulmonary disease or significant valvular disease might cause frank respiratory insufficiency. Likewise, any fall in blood pressure might cause cerebral ischaemia in a patient with significant cerebral vascular disease or might cause profound hypotension in a patient with significant aortic stenosis. Other parameters to consider include the complexity of the disease, interventional procedures/techniques, and the clinical presentation of the patient. For example, a systolic blood pressure/LVEDP ratio of <4 has been proven to predict mortality better than LVEDP alone in STEMI patients.^44^ Accordingly, we propose the algorithm outlined in *Figure 2*, which we acknowledge awaits validation in a large cohort of patients. As proposed in *Figure 2A*, MCS support as a standby option must be critically scrutinized in light of recent data^39,40,42^ and perhaps an algorithm without MCS as a standby option, outlined in *Figure 2B*, might be more appropriate but requires further investigation. Of course, it is unclear whether an MCS standby option with intravascular sheaths or a preemtively placed wire in the LV will allow for faster implementation of support in the case of haemodynamic collapse could also be an appropriate third option.

## Conclusion

There remain some discrepancies in the results of small-scale studies, registries, and the results of randomized clinical trials regarding the benefits of MCS in HR-PCI. Moreover, the data on IABP and ECMO use remain sparse or out of date (particularly for IABP), thereby not accurately reflecting contemporary interventional practices. The most abundant and recent data available are for the Impella device, indicating that Impella provides even greater benefits in HR-PCI than outlined in the initial PROTECT II study published more than a decade ago. These benefits are likely due to improved handling and technical advancements of the current available Impella devices, which lead to fewer bleeding complications and favour more complete revascularization. The PROTECT IV randomized clinical trial aims to clarify these important questions for the use of Impella in HR-PCI.

## Lead author biography

Dr Alexander Geppert received his doctorate of medicine from the Medical University of Vienna in March 1991. He has speciality training in internal medicine, intensive care, and cardiology. In June 2003, he became Professor of Internal Medicine at the Medical University of Vienna and Director of the Intensive Care Unit at the Medicine Clinic Ottakring from 2003 to 2014, Director of Catheterization Laboratory from 2014 to 2022, and past chair of the working group of interventional cardiology of the Austrian Society of Cardiology. Currently, he serves as the Director of CHIP/CTO/Impella and TAVI programme and is the coordinator of the Austrian CTO Network.

## References

[1] Velazquez EJ, Lee KL, Jones RH, Al-Khalidi HR, Hill JA, Panza JA, Michler RE, Bonow RO, Doenst T, Petrie MC, Oh JK, She L, Moore VL, Desvigne-Nickens P, Sopko G, Rouleau JL; STICHES Investigators. Coronary-artery bypass surgery in patients with ischemic cardiomyopathy. N Engl J Med 2016;374:1511–1520. 27040723 10.1056/NEJMoa1602001 PMC4938005

[2] Mohr FW, Morice MC, Kappetein AP, Feldman TE, Ståhle E, Colombo A, Mack MJ, Holmes DR Jr, Morel MA, Van Dyck N, Houle VM, Dawkins KD, Serruys PW. Coronary artery bypass graft surgery versus percutaneous coronary intervention in patients with three-vessel disease and left main coronary disease: 5-year follow-up of the randomised, clinical SYNTAX trial. Lancet 2013;381:629–638. 23439102 10.1016/S0140-6736(13)60141-5

[3] Head SJ, Milojevic M, Daemen J, Ahn JM, Boersma E, Christiansen EH, Domanski MJ, Farkouh ME, Flather M, Fuster V, Hlatky MA, Holm NR, Hueb WA, Kamalesh M, Kim YH, Mäkikallio T, Mohr FW, Papageorgiou G, Park SJ, Rodriguez AE, Sabik JF III, Stables RH, Stone GW, Serruys PW, Kappetein AP. Mortality after coronary artery bypass grafting versus percutaneous coronary intervention with stenting for coronary artery disease: a pooled analysis of individual patient data. Lancet 2018;391:939–948. 29478841 10.1016/S0140-6736(18)30423-9

[4] Salisbury AC, Kirtane AJ, Ali ZA, Grantham JA, Lombardi WL, Yeh RW, Genereux P, Allen KB, Brown WM, Nugent K, Gosch KL, Karmpaliotis D, Spertus JA, Kandzari DE; OPTIMUM Study Investigators. The Outcomes of Percutaneous RevascularizaTIon for Management of SUrgically Ineligible Patients with Multivessel or Left Main Coronary Artery Disease (OPTIMUM) registry: rationale and design. Cardiovasc Revasc Med 2022;41:83–91. 35120846 10.1016/j.carrev.2022.01.008

[5] Chieffo A, Burzotta F, Pappalardo F, Briguori C, Garbo R, Masiero G, Nicolini E, Ribichini F, Trani C, Álvarez BC, Leor OR, Moreno R, Santos R, Fiarresga A, Silveira JB, de Prado AP, Musumeci G, Esposito G, Tarantini G. Clinical expert consensus document on the use of percutaneous left ventricular assist support devices during complex high-risk indicated PCI: Italian Society of Interventional Cardiology working group endorsed by Spanish and Portuguese interventional cardiology societies. Int J Cardiol 2019;293:84–90. 31174920 10.1016/j.ijcard.2019.05.065

[6] Rihal CS, Naidu SS, Givertz MM, Szeto WY, Burke JA, Kapur NK, Kern M, Garratt KN, Goldstein JA, Dimas V, Tu T; Society for Cardiovascular Angiography and Interventions (SCAI); Heart Failure Society of America (HFSA); Society of Thoracic Surgeons (STS); American Heart Association (AHA), and American College of Cardiology (ACC). 2015 SCAI/ACC/HFSA/STS clinical expert consensus statement on the use of percutaneous mechanical circulatory support devices in cardiovascular care: endorsed by the American Heart Assocation, the Cardiological Society of India, and Sociedad Latino Americana de Cardiologia Intervencion; affirmation of value by the Canadian Association of Interventional Cardiology-Association Canadienne de Cardiologie d’intervention. J Am Coll Cardiol 2015;65:e7–e26. 25861963 10.1016/j.jacc.2015.03.036

[7] Sandhu A, McCoy LA, Negi SI, Hameed I, Atri P, Al’Aref SJ, Curtis J, McNulty E, Anderson HV, Shroff A, Menegus M, Swaminathan RV, Gurm H, Messenger J, Wang T, Bradley SM. Use of mechanical circulatory support in patients undergoing percutaneous coronary intervention: insights from the National Cardiovascular Data Registry. Circulation 2015;132:1243–1251. 26286905 10.1161/CIRCULATIONAHA.114.014451

[8] Flynn MS, Kern MJ, Donohue TJ, Aguirre FV, Bach RG, Caracciolo EA. Alterations of coronary collateral blood flow velocity during intraaortic balloon pumping. Am J Cardiol 1993;71:1451–1455. 8517395 10.1016/0002-9149(93)90611-f

[9] Parissis H, Graham V, Lampridis S, Lau M, Hooks G, Mhandu PC. IABP: history-evolution-pathophysiology-indications: what we need to know. J Cardiothorac Surg 2016;11:122. 27487772 10.1186/s13019-016-0513-0 PMC4972967

[10] Lindholm JA . Cannulation for veno-venous extracorporeal membrane oxygenation. J Thorac Dis 2018;10:S606–S612. 29732177 10.21037/jtd.2018.03.101 PMC5911563

[11] Aurigemma C, Burzotta F, Chieffo A, Briguori C, Piva T, De Marco F, Di Biasi M, Pagnotta P, Casu G, Garbo R, Trani C, Tarantini G; IMP-IT Investigators. Clinical impact of revascularization extent in patients undergoing Impella-protected PCI enrolled in a nationwide registry. JACC Cardiovasc Interv 2021;14:717–719. 33736787 10.1016/j.jcin.2021.01.017

[12] Burke DA, Kundi H, Almonacid A, O’Neill W, Moses J, Kleiman N, Dixon S, Palacios I, Guzman LA, Ohman EM, Popma JJ, Pinto DS. The value of left ventricular support in patients with reduced left ventricular function undergoing extensive revascularization: an analysis from the PROTECT-II randomized trial. JACC Cardiovasc Interv 2019;12:1985–1987. 31601396 10.1016/j.jcin.2019.07.050

[13] Wollmuth J, Patel MP, Dahle T, Bharadwaj A, Waggoner TE, Chambers JW, Ruiz-Rodriguez E, Mahmud E, Thompson C, Morris DL; RESTORE EF Investigators. Ejection fraction improvement following contemporary high-risk percutaneous coronary intervention: RESTORE EF study results. J Soc Cardiovasc Angiogr Interv 2022;1:100350. 10.1016/j.jscai.2022.100350 PMC11307872 39131473

[14] Buccheri S, Franchina G, Romano S, Puglisi S, Venuti Ge, D’Arrigo P, Francaviglia B, Scalia M, Condorelli A, Barbanti M, Capranzano P, Tamburino C, Capodanno D. Clinical outcomes following intravascular imaging-guided versus coronary angiography-guided percutaneous coronary intervention with stent implantation: a systematic review and Bayesian network meta-analysis of 31 studies and 17,882 patients. JACC Cardiovasc Interv 2017;10:2488–2498. 29153502 10.1016/j.jcin.2017.08.051

[15] Meneveau N, Souteyrand G, Motreff P, Caussin C, Amabile N, Ohlmann P, Morel O, Lefrançois Y, Descotes-Genon V, Silvain J, Braik N, Chopard R, Chatot M, Ecarnot F, Tauzin H, Van Belle E, Belle L, Schiele F. Optical coherence tomography to optimize results of percutaneous coronary intervention in patients with non-ST-elevation acute coronary syndrome: results of the multicenter, randomized DOCTORS study (Does Optical Coherence Tomography Optimize Results of Stenting). Circulation 2016;134:906–917. 27573032 10.1161/CIRCULATIONAHA.116.024393

[16] Mintz GS, Bourantas CV, Chamié D. Intravascular imaging for percutaneous coronary intervention guidance and optimization: the evidence for improved patient outcomes. J Soc Cardiovasc Angiogr Interv 2022;1:100413. 10.1016/j.jscai.2022.100413 PMC11307675 39132365

[17] Lee JM, Choi KH, Song YB, Lee JY, Lee SJ, Lee SY, Kim SM, Yun KH, Cho JY, Kim CJ, Ahn HS, Nam CW, Yoon HJ, Park YH, Lee WS, Jeong JO, Song PS, Doh JH, Jo SH, Yoon CH, Kang MG, Koh JS, Lee KY, Lim YH, Cho YH, Cho JM, Jang WJ, Chun KJ, Hong D, Park TK, Yang JH, Choi SH, Gwon HC, Hahn JY; RENOVATE-COMPLEX-PCI Investigators. Intravascular imaging-guided or angiography-guided complex PCI. N Engl J Med 2023;388:1668–1679. 36876735 10.1056/NEJMoa2216607

[18] O’Neill WW, Kleiman NS, Moses J, Henriques JPS, Dixon S, Massaro J, Palacios I, Maini B, Mulukutla S, Dzavík V, Popma J, Douglas PS, Ohman M. A prospective, randomized clinical trial of hemodynamic support with Impella 2.5 versus intra-aortic balloon pump in patients undergoing high-risk percutaneous coronary intervention: the PROTECT II study. Circulation 2012;126:1717–1727. 22935569 10.1161/CIRCULATIONAHA.112.098194

[19] Thiele H, Jobs A, Ouweneel DM, Henriques JPS, Seyfarth M, Desch S, Eitel I, Pöss J, Fuernau G, de Waha S. Percutaneous short-term active mechanical support devices in cardiogenic shock: a systematic review and collaborative meta-analysis of randomized trials. Eur Heart J 2017;38:3523–3531. 29020341 10.1093/eurheartj/ehx363

[20] Fearon WF, Nishi T, De Bruyne B, Boothroyd DB, Barbato E, Tonino P, Jüni P, Pijls NHJ, Hlatky MA; FAME 2 Trial Investigators. Clinical outcomes and cost-effectiveness of fractional flow reserve-guided percutaneous coronary intervention in patients with stable coronary artery disease. Circulation 2018;137:480–487. 29097450 10.1161/CIRCULATIONAHA.117.031907

[21] Perera D, Stables R, Thomas M, Booth J, Pitt M, Blackman D, de Belder A, Redwood S; BCIS-1 Investigators. Elective intra-aortic balloon counterpulsation during high-risk percutaneous coronary intervention: a randomized controlled trial. JAMA 2010;304:867–874. 20736470 10.1001/jama.2010.1190

[22] Perera D, Stables R, Clayton T, De Silva K, Lumley M, Clack L, Thomas M, Redwood S; BCIS-1 Investigators. Long-term mortality data from the balloon pump-assisted coronary intervention study (BCIS-1). Circulation 2013;127:207–212. 23224207 10.1161/CIRCULATIONAHA.112.132209

[23] Romeo F, Acconcia MC, Sergi D, Romeo A, Gensini GF, Chiarotti F, Caretta Q. Lack of intra-aortic balloon pump effectiveness in high-risk percutaneous coronary interventions without cardiogenic shock: a comprehensive meta-analysis of randomised trials and observational studies. Int J Cardiol 2013;167:1783–1793. 23295041 10.1016/j.ijcard.2012.12.027

[24] Chieffo A, Dudek D, Hassager C, Combes A, Gramegna M, Halvorsen S, Huber K, Kunadian V, Maly J, Møller JE, Pappalardo F, Tarantini G, Tavazzi G, Thiele H, Vandenbriele C, van Mieghem N, Vranckx P, Werner N, Price S. Joint EAPCI/ACVC expert consensus document on percutaneous ventricular assist devices. Eur Heart J Acute Cardiovasc Care 2021;10:570–583. 34057173 10.1093/ehjacc/zuab015 PMC8245145

[25] van den Brink FS, Meijers TA, Hofma SH, van Boven AJ, Nap A, Vonk A, Symersky P, Sjauw KD, Knaapen P. Prophylactic veno-arterial extracorporeal membrane oxygenation in patients undergoing high-risk percutaneous coronary intervention. Neth Heart J 2020;28:139–144. 31782108 10.1007/s12471-019-01350-8 PMC7052097

[26] Tomasello SD, Boukhris M, Ganyukov V, Galassi AR, Shukevich D, Haes B, Kochergin N, Tarasov R, Popov V, Barbarash L. Outcome of extracorporeal membrane oxygenation support for complex high-risk elective percutaneous coronary interventions: a single-center experience. Heart Lung 2015;44:309–313. 25913808 10.1016/j.hrtlng.2015.03.005

[27] Marchese A, Tarantini G, Tito A, Margari V, Resta F, Dhojniku I, Paparella D, Speziale G. Mechanical circulatory support and intravascular lithotripsy in high-risk patients undergoing percutaneous coronary intervention and transcatheter aortic valve replacement: a case series. Eur Heart J Case Rep 2021;5:ytab498. 35047739 10.1093/ehjcr/ytab498 PMC8759477

[28] Zhao L, Zhang CP, Wang HR, Li ZB, Liu B. Efficacy of extracorporeal membrane oxygenation in patients with complex high risk coronary artery disease undergoing percutaneous coronary intervention. Zhonghua Xin Xue Guan Bing Za Zhi 2021;49:757–763. 34404183 10.3760/cma.j.cn112148-20210324-00270

[29] O’Neill WW, Anderson M, Burkhoff D, Grines CL, Kapur NK, Lansky AJ, Mannino S, McCabe JM, Alaswad K, Daggubati R, Wohns D, Meraj PM, Pinto DS, Popma JJ, Moses JW, Schreiber TL, Magnus Ohman E. Improved outcomes in patients with severely depressed LVEF undergoing percutaneous coronary intervention with contemporary practices. Am Heart J 2022;248:139–149. 35192839 10.1016/j.ahj.2022.02.006

[30] Cohen MG, Matthews R, Maini B, Dixon S, Vetrovec G, Wohns D, Palacios I, Popma J, Ohman EM, Schreiber T, O’Neill WW. Percutaneous left ventricular assist device for high-risk percutaneous coronary interventions: real-world versus clinical trial experience. Am Heart J 2015;170:872–879. 26542494 10.1016/j.ahj.2015.08.009

[31] Burzotta F, Russo G, Ribichini F, Piccoli A, D’Amario D, Paraggio L, Previ L, Pesarini G, Porto I, Leone AM, Niccoli G, Aurigemma C, Verdirosi D, Crea F, Trani C. Long-term outcomes of extent of revascularization in complex high risk and indicated patients undergoing Impella-protected percutaneous coronary intervention: report from the Roma-Verona registry. J Interv Cardiol 2019;2019:5243913. 31772533 10.1155/2019/5243913 PMC6739781

[32] Kovacic JC, Kini A, Banerjee S, Dangas G, Massaro J, Mehran R, Popma J, O’Neill WW, Sharma SK. Patients with 3-vessel coronary artery disease and impaired ventricular function undergoing PCI with Impella 2.5 hemodynamic support have improved 90-day outcomes compared to intra-aortic balloon pump: a sub-study of the PROTECT II trial. J Interv Cardiol 2015;28:32–40. 25689546 10.1111/joic.12166

[33] Henriques JP, Ouweneel DM, Naidu SS, Palacios IF, Popma J, Ohman EM, O’Neill WW. Evaluating the learning curve in the prospective randomized clinical trial of hemodynamic support with Impella 2.5 versus intra-aortic balloon pump in patients undergoing high-risk percutaneous coronary intervention: a prespecified subanalysis of the PROTECT II study. Am Heart J 2014;167:472–479.e5. 24655695 10.1016/j.ahj.2013.12.018

[34] Flaherty MP, Moses JW, Westenfeld R, Palacios I, O’Neill WW, Schreiber TL, Lim MJ, Kaki A, Ghiu I, Mehran R. Impella support and acute kidney injury during high-risk percutaneous coronary intervention: the Global cVAD Renal Protection Study. Catheter Cardiovasc Interv 2020;95:1111–1121. 31355987 10.1002/ccd.28400

[35] Flaherty MP, Pant S, Patel SV, Kilgore T, Dassanayaka S, Loughran JH, Rawasia W, Dawn B, Cheng A, Bartoli CR. Hemodynamic support with a microaxial percutaneous left ventricular assist device (Impella) protects against acute kidney injury in patients undergoing high-risk percutaneous coronary intervention. Circ Res 2017;120:692–700. 28073804 10.1161/CIRCRESAHA.116.309738

[36] Schweitzer J, Horn P, Voss F, Kivel M, Wolff G, Jung C, Zeus T, Kelm M, Westenfeld R. Incidence of acute kidney injury is lower in high-risk patients undergoing percutaneous coronary intervention supported with Impella compared to ECMO. J Cardiovasc Transl Res 2022;15:239–248. 34324156 10.1007/s12265-021-10141-9 PMC8983546

[37] Song Z, Wang C, Stammers AH. Clinical comparison of pulsatile and nonpulsatile perfusion during cardiopulmonary bypass. J Extra Corpor Technol 1997;29:170–175. 10176125

[38] Van Den Buijs DMF, Wilgenhof A, Knaapen P, Zivelonghi C, Meijers T Vermeersch P, Arslan F, Verouden N, Nap A, Sjauw K, van den Brink FS. Prophylactic Impella CP versus VA-ECMO in patients undergoing complex high-risk indicated PCI. J Interv Cardiol 2022;2022:8167011. 36447936 10.1155/2022/8167011 PMC9663242

[39] O’Neill BP, Grines C, Moses JW, Ohman EM, Lansky A, Popma J, Kapur NK, Schreiber T, Mann’no S, O’Neill WW, Medjamia AM, Mahmud E. Outcomes of bailout percutaneous ventricular assist device versus prophylactic strategy in patients undergoing nonemergent percutaneous coronary intervention. Catheter Cardiovasc Interv 2021;98:E501–E512. 34051033 10.1002/ccd.29758

[40] Tarantini G, Masiero G, Burzotta F, Pazzanese V, Briguori C, Trani C, Piva T, De Marco F, Di Biasi M, Pagnotta P, Mojoli M, Casu G, Giustino G, Lorenzoni G, Montorfano M, Ancona MB, Pappalardo F, Chieffo A; IMPella Mechanical Circulatory Support Device in Italy (IMP-IT) Registry authors. Timing of Impella implantation and outcomes in cardiogenic shock or high-risk percutaneous coronary revascularization. Catheter Cardiovasc Interv 2021;98:E222–E234. 33793051 10.1002/ccd.29674 PMC8451815

[41] Briguori C, Sarais C, Pagnotta P, Airoldi F, Liistro F, Sgura F, Spanos V, Carlino M, Montorfano M, Di Mario C, Colombo A. Elective versus provisional intra-aortic balloon pumping in high-risk percutaneous transluminal coronary angioplasty. Am Heart J 2003;145:700–707. 12679768 10.1067/mhj.2003.14

[42] Danek BA, Basir MB, O’Neill WW, Alqarqaz M, Karatasakis A, Karmpaliotis D, Jaffer FA, Yeh RW, Wyman M, Lombardi WL, Kandzari D, Lembo N, Doing A, Patel M, Mahmud E, Choi JW, Toma C, Moses JW, Kirtane A, Parikh M, Ali ZA, Garcia S, Karacsonyi J, Rangan BV, Thompson CA, Banerjee S, Brilakis ES, Alaswad K. Mechanical circulatory support in chronic total occlusion percutaneous coronary intervention: insights from a multicenter U.S. Registry. J Invasive Cardiol 2018;30:81–87. 29493509

[43] Al-Rashid F, Mahabadi AA, Johannsen L, Soldat J, Dykun I, Jánosi RA, Totzeck M, Rassaf T. Impact of left-ventricular end-diastolic pressure as a predictor of periprocedural hemodynamic deterioration in patients undergoing Impella supported high-risk percutaneous coronary interventions. Int J Cardiol Heart Vasc 2020;26:100445. 31799370 10.1016/j.ijcha.2019.100445 PMC6881640

[44] Sola M, Venkatesh K, Caughey M, Rayson R, Dai X, Stouffer GA, Yeung M. Ratio of systolic blood pressure to left ventricular end-diastolic pressure at the time of primary percutaneous coronary intervention predicts in-hospital mortality in patients with ST-elevation myocardial infarction. Catheter Cardiovasc Interv 2017;90:389–395. 28303647 10.1002/ccd.26963

